# Measuring the in-hospital costs of *Pseudomonas aeruginosa* pneumonia: methodology and results from a German teaching hospital

**DOI:** 10.1186/s12879-019-4660-5

**Published:** 2019-12-03

**Authors:** Klaus Kaier, Thomas Heister, Tim Götting, Martin Wolkewitz, Nico T. Mutters

**Affiliations:** 1 Institute of Medical Biometry and Statistics, Faculty of Medicine and Medical Center, University of Freiburg, Freiburg, Germany; 2 Institute for Infection Prevention and Hospital Epidemiology, Faculty of Medicine and Medical Center, University of Freiburg, Freiburg, Germany

**Keywords:** *P. aeruginosa* pneumonia, Community-acquired infection, Hospital-acquired infection, Statistical methods, Time-dependent exposure

## Abstract

**Background:**

*Pseudomonas aeruginosa-*related pneumonia is an ongoing healthcare challenge. Estimating its financial burden is complicated by the time-dependent nature of the disease.

**Methods:**

Two hundred thirty-six cases of *Pseudomonas aeruginosa-*related pneumonia were recorded at a 2000 bed German teaching hospital between 2011 and 2014. Thirty-five cases (15%) were multidrug-resistant (MDR) *Pseudomonas aeruginosa*. Hospital- and community-acquired cases were distinguished by main diagnoses and exposure time. The impact of *Pseudomonas aeruginosa*-related pneumonia on the three endpoints cost, reimbursement, and length of stay was analyzed, taking into account (1) the time-dependent nature of exposure, (2) clustering of costs within diagnostic groups, and (3) additional confounders.

**Results:**

*Pseudomonas aeruginosa* pneumonia is associated with substantial additional costs that are not fully reimbursed. Costs are highest for hospital-acquired cases (€19,000 increase over uninfected controls). However, community-acquired cases are also associated with a substantial burden (€8400 when *Pseudomonas aeruginosa* pneumonia is the main reason for hospitalization, and €6700 when not). Sensitivity analyses for hospital-acquired cases showed that ignoring or incorrectly adjusting for time-dependency substantially biases results. Furthermore, multidrug-resistance was rare and only showed a measurable impact on the cost of community-acquired cases.

**Conclusions:**

*Pseudomonas aeruginosa* pneumonia creates a substantial financial burden for hospitals. This is particularly the case for nosocomial infections. Infection control interventions could yield significant cost reductions. However, to evaluate the potential effectiveness of different interventions, the time-dependent aspects of incremental costs must be considered to avoid introduction of bias.

## Background

Pneumonia presents an ongoing healthcare challenge. Community-acquired cases account for a considerable number of hospitalizations [1, 2], and hospital-acquired pneumonia is a common complication in both ventilated [3] and non-ventilated [4] patients. Hospital-acquired cases of pneumonia are furthermore associated with increased total hospital expenditure, longer length of stay, and greater likelihood of death [5, 6].

A variety of causative pathogens have been identified, depending on the patient population and other factors [7–11]. However, a relatively limited set of bacterial species have been identified as causing a large number of the hospital-acquired bacterial cases, i.e. *Staphylococcus aureus*, *Pseudomonas aeruginosa*, *Klebsiella* species, *Escherichia coli*, *Acinetobacter* species, and *Enterobacter* species [7].

*Pseudomonas aeruginosa* is not only a frequent causative agent of pneumonia in hospitalized patients [12], immunocompromised hosts, and patients with cystic fibrosis [13], it is also a common cause of community-acquired infection [14] and is responsible for considerable additional healthcare costs and resource utilization [12]. However, assessing the cost of an infection such as pneumonia is prone to biases. Multidrug-resistance (MDR) introduces additional difficulty and might be an important variable in explaining the burden of pneumonia.

Precise measurement of infection-associated costs is vital for hospital boards and administrators to guide investment and budgeting decisions and long-term planning of structural and non-structural infection control measures. Lastly, it is also crucial for policy makers and health insurance providers.

This study demonstrates appropriate analysis strategies using real clinical data and estimates the economic burden of both hospital-acquired and community-acquired pneumonia, taking into account exposure time, clustering of costs within procedural groups, and multidrug-resistance. Using *Pseudomonas aeruginosa-*related pneumonia as an example, this study also discusses the methodological challenges of estimating the hospital costs associated with infections.

## Methods

When analyzing the hospital costs attributable to a specific pathogen and condition such as *Pseudomonas aeruginosa-*related pneumonia, a number of methodological issues need to be kept in mind. Firstly, three categories of cases should be differentiated when estimating the cost of *Pseudomonas aeruginosa-*related pneumonia [15]:

1. Hospital-acquired pneumonia (HAP), where pneumonia is detected more than 48 h after admission and by definition is not the main reason for hospitalization. To estimate the additional cost of HAPs, three particular aspects need to be kept in mind. First, in-hospital costs are highly clustered within diagnostic groups due to the high share of disease- and procedure-related fixed costs. Secondy, the time-dependent nature of healthcare-associated infections (HAI) needs to be taken into account to avoid an overestimation of the true effect due to time-dependent bias [16–18]. Thirdly, the impact of HAI on the costs of care may be confounded by other cost drivers such as advanced age and comorbidities.
2. Community-acquired pneumonia cases (CAP), where the infection is present upon hospital admission, but not the primary reason for hospitalization. Here, the additional costs of the infection are calculated by comparing these cases to uninfected controls.
3. CAPs in which the infection **is** the main reason for hospitalization (e.g. main diagnosis group = *Pseudomonas aeruginosa-*related pneumonia). These cases pose little analytical challenge, as the (additional) costs are identical to the total cost of the hospital stay since the patient would not have been hospitalized in the first place if not for the infection.

Knowing the point in time when exposure took place is crucial. If it is unknown, it is impossible to distinguish between the three categories of infections outlined above. Analyzing the excess cost of treating HAPs without taking into account the timing of HAP onset causes the results to be subject to time-dependent bias [18]. However, the exact exposure time often cannot be gleaned from routinely collected clinical data [4, 12]. Given this limitation, some authors have proposed matching (or adjusting) for total length of stay [12, 19]. This approach, however, is problematic as it is subject to “conditioning on the future”, i.e. controlling for an outcome [20, 21]. Essentially, this is a statistical variant of the hindsight bias by predicting an outcome based on information influenced by the outcome itself [22].

Another challenge concerns the identification of appropriate controls. The clustering of costs within procedural groups should be accounted for [23], and only comorbidities which cannot plausibly occur as a consequence of an infection should be used for risk adjustment to prevent controlling for an outcome [24, 25].

We investigated the issues outlined above in clinical data collected from a 2000 bed German University teaching hospital between 2011 and 2014. Consisting of 204,914 complete patient records, the data included information on age, gender, main and secondary diagnoses, and cost figures calculated according to the standardized costing system developed by the Institute for the Hospital Remuneration System (InEK), the authority responsible for reimbursement rates [26, 27]. All patient data was anonymized in accordance with German law. Written consent was thus determined to not be necessary. The study’s use of the data was approved by the University of Freiburg Ethics committee.

Cases of *Pseudomonas aeruginosa-*related pneumonia were identified using the main or secondary diagnosis J.151 (ICD-10). Next, cases were confirmed and/or categorized using additional information regarding the microbiological and clinical details of the respective pathogen. The exact time at which exposure took place (since admission) is available for all confirmed cases of *Pseudomonas aeruginosa-*related pneumonia. *Pseudomonas aeruginosa*-related pneumonia cases are categorized by the place of acquisition (outside the hospital/inside the hospital) and the immunity of the patient (immune competence). The status of a *Pseudomonas aeruginosa*-related pneumonia case was only applied to patients who fulfill the epidemiological, microbiological and clinical criteria for *Pseudomonas aeruginosa*-related pneumonia in accordance to the relevant German guidelines [28, 29].

We calculated the burden of *Pseudomonas aeruginosa-*related pneumonia for (1) HAP, (2) CAP where *Pseudomonas aeruginosa-*related pneumonia was *not* the main reason for hospitalization, and (3) CAP where *Pseudomonas aeruginosa-*related pneumonia *was* the main reason for hospitalization. In each of the following steps and scenarios, the three endpoints cost, reimbursement, and length of stay were analyzed using quantile regression to account for the right skewed nature of the data. Quantile regression does not require distributional assumptions other than continuity of the dependent variable, and the resulting estimates are considered completely robust to extreme values of the dependent variable [30]. All analyses were conducted using Stata 15 (StataCorp, College Station, TX, USA). We developed the following 5-step approach:

### Analysis of HAP

We used regression models with a within-main-diagnosis time-to-exposure stratification approach that allows for appropriate treatment of the time-dependent exposure, while also accounting for the clustering of costs within main diagnosis groups. Each case of HAP was matched with up to four controls within the same main diagnosis groups that had a length of stay at least equal to the exposure time of each case. Time-to-exposure matching has previously been suggested as a suitable way to analyze the additional cost of hospital-acquired infections [31]. In the regression analyses, the strata consisting of the case and the four matched controls have been added as fixed effects. Baseline risks are controlled for by adding the Charlson Comorbidity Index (CCI), sex, age, and age^2^ as further covariates.

### Analysis of CAP where pneumonia was *not* the main reason for hospitalization

We used a within-main-diagnosis regression approach that allows for a comparison of patients with a similar risk and cost propensity on admission. Regression specification and risk adjustment are analog to the model described above, merely without the time-to-exposure component, since the infection is already present on admission.

### Analysis of CAP where pneumonia *was* the main reason for hospitalization

As mentioned, these cases pose little analytical challenge, as the patient would not have been hospitalized had it not been for the infection and the (additional) costs thus are identical to the total costs of the hospital stay.

### Potential biases from misspecification of HAP analyses

Analyses of the incremental costs of hospital-acquired conditions are prone to time-related bias if the timing of exposure is neglected [32]. We therefore developed two sensitivity analyses. First, we presented a sensitivity analysis in which the time-dependency of exposure in HAP is ignored in order to quantify the potential overestimation of the true effect [33]. Next we conducted an analysis that was done when the exposure time was still unavailable [19, 34]. Specifically, we again ignored the time-dependency of exposure, and rather adjusted for total length of stay.

### Impact of multidrug-resistance (MDR)

The burden of *Pseudomonas aeruginosa* pneumonia was stratified for MDR. Analyses in Step (I), (II), and (III) were repeated, but resistance status (MDR or non-MDR *Pseudomonas aeruginosa*) was included in the regression model as an effect modifier. This approach means that different types of infections (MDR or non-MDR *Pseudomonas aeruginosa*) were compared to uninfected controls. Previous studies often directly compared MDR and non-MDR cases [35], which implies the assumption of the so-called “replacement scenario”: It assumes that every infection caused by resistant bacteria would be replaced by an infection caused by more susceptible bacteria if the spread of resistant pathogens was prevented [36–38]. Yet antibiotic resistance does not only increase the burden of infections: It is also responsible for the onset of infection through the failure of antibiotic prophylaxis [39].

## Results

**Fig. 1:**
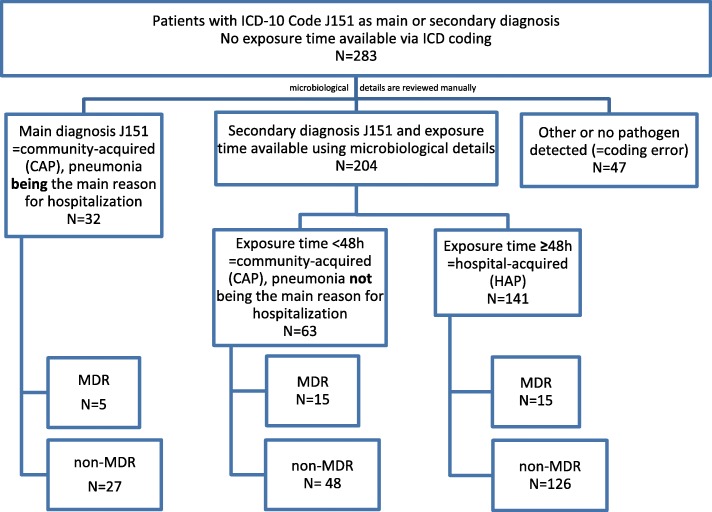
Patient selection and categorization of *Pseudomonas aeruginosa* related pneumonia

As shown in Fig. 1, a total of 283 cases of *Pseudomonas aeruginosa*-related pneumonia were recorded as main or secondary diagnoses. Microbiological review confirmed 236 (83%) cases. Only 35 (15%) cases were MDR *Pseudomonas aeruginosa*.

**Fig. 2:**
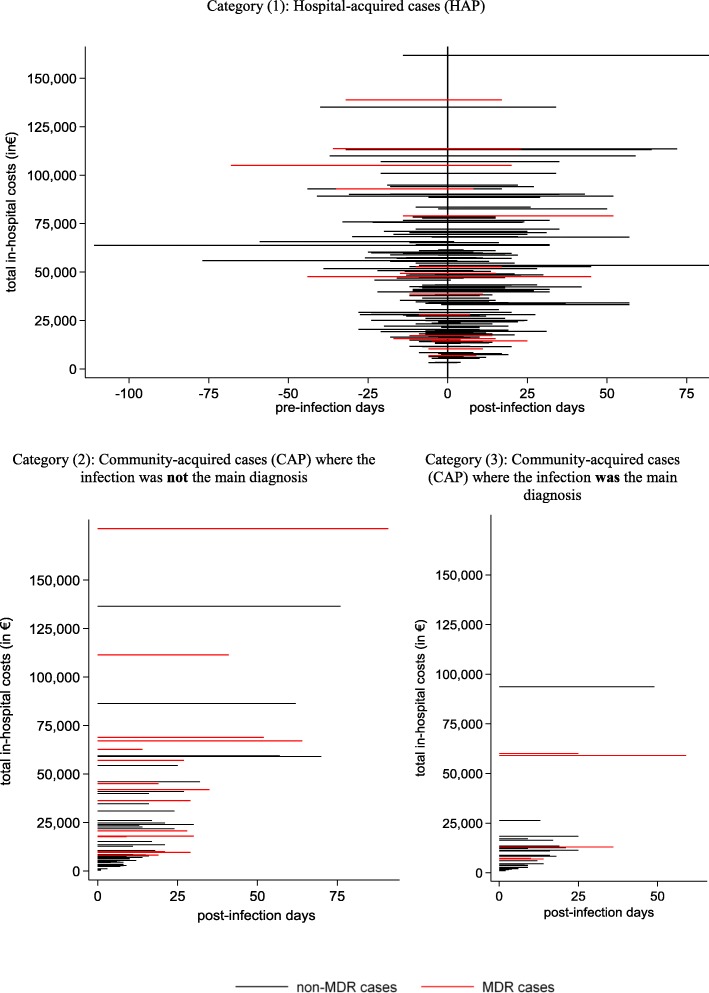
Hospital and community-acquired cases of *Pseudomonas aeruginosa* related pneumonia

The data included 141 cases of HAP, which on average were associated with €46,500 in costs and €40,500 in reimbursements (see Table 1). Furthermore, 63 cases of CAP were identified where pneumonia was *not* the primary reason for hospitalization. The average costs and reimbursements for these cases were €27,000 and €23,000, respectively. Cases where CAP *was* the main reason for hospitalization were associated with €14,000 in both costs and reimbursements. The differences in costs are reflected in the average length of stay for the three groups of 35, 21 and 15 days, respectively. As shown in Fig. 2, these mean values are to some extent driven by outliers, which results in a substantial difference between the means and medians.

**Table 1:** Descriptive Statistics for cases of *Pseudomonas aeruginosa* pneumonia

	Hospital-acquired cases (HAP)		Community-acquired cases where the infection was not the main diagnosis (CAP)		Community-acquired cases where the infection was the main diagnosis (CAP)	
	Mean	SD	Mean	SD	Mean	SD
Costs (in €)	46,542.7	31,624.2	26,954.9	33,540.6	14,065.8	20,177.4
Reimbursement (in €)	40,453.5	29,537.1	23,122.0	28,216.1	13,712.7	22,011.3
Length of stay (in days)	35.95	22.78	21.13	19.15	15.47	12.74
Exposure time (in days)	24.28	95.91	0.84	2.86	4.25	15.49
Charlson Comorbidity Index (CCI)	4.01	3.00	3.57	3.09	2.38	2.69
Deaths (in %)	23%		21%		12%	
Age (in years)	65.22	14.51	59.00	15.63	61.72	19.82
Female gender (in %)	28%		30%		56%	
N	141		63		32	

Details on patients with *Pseudomonas aeruginosa* pneumonia which were confirmed by microbiological review at a German University teaching hospital between 2011 and 2014. Categorization according to main diagnosis and exposure time (HAP: detected more than 48 h after admission)

Results from regression analyses presented in Table 2 column 1 show that HAP cases are associated with additional costs of €19,000 and reimbursements of €17,000 compared with uninfected controls.

**Table 2:** Additional burden of hospital-acquired cases of *Pseudomonas aeruginosa* pneumonia

	(1)	(2)	(3)
Additional costs (in €)	19,019.6^***^	30,453.1^***^	12,289.4^***^
	[12,944.3, 250,94.9]	[22,418.4, 38,487.8]	[5400.6, 19,178.2]
Additional reimbursement (in €)	16,599.9^***^	29,401.4^***^	7811.5^**^
	[10,025.9, 23,173.8]	[22,536.2, 36,266.6]	[1694.5, 13,928.5]
Additional length of stay (in days)	9.141^***^	18.75^***^	
	[6.941, 11.34]	[16.20, 21.30]	
Time-to exposure matching	yes	no	no
Adjusting for total length of stay	no	no	yes
N	555	584	584

95% confidence intervals in brackets, results of median regression

^*^ *p* < 0.1, ^**^ *p* < 0.05, ^***^ *p* < 0.01

Results of multivariate quantile regressions including a case-control variable as well as CCI, sex, age, and age^2^ as fixed effects. Column (1) shows the ‘correct’ model, whereas column (2) shows the effect of ignoring the time dependency of exposure. Column (3) shows the results of a regression controlling for overall length of stay instead of matching on the time of infection

As shown in Table 3, CAP where the pneumonia was *not* the primary reason for hospital admission incurred additional costs of about €6700, of which only €5400 were compensated through higher reimbursement.

**Table 3:** Additional burden of community-acquired cases where the infection was not the main diagnosis

Additional costs (in €)	6689.1^***^
	[4368.3, 9009.9]
Additional reimbursement (in €)	5354.3^***^
	[2152.3, 8556.3]
Additional length of stay (in days)	7.264^***^
	[3.996, 10.53]
*N*	262

95% confidence intervals in brackets

^*^ *p* < 0.1, ^**^ *p* < 0.05, ^***^ *p* < 0.01

Results of multivariate quantile regressions including a case-control variable as well as CCI, sex, age, and age^2^ as fixed effects

Table 4 shows that cases where CAP *was* the primary reason for hospitalization incurred a (median) total cost of about €8400 and reimbursement of €5400. Since *Pseudomonas aeruginosa* pneumonia is the reason for hospital admission in the latter case, these costs represent the burden from the hospital perspective, as the patient would not otherwise have been admitted.

**Table 4:** Burden of community-acquired cases where the infection was the main diagnosis

Total costs (in €)	8377.3^***^
	[4330.8, 12,423.7]
Total reimbursement (in €)	5442.6***
	[2287.8, 8597.3]
Total length of stay (in days)	13.0^***^
	[8.876, 17.12]
*N*	32

95% confidence intervals in brackets

^*^ *p* < 0.1, ^**^ *p* < 0.05, ^***^ *p* < 0.01

Results of univariate quantile regression

Table 2 also shows the effect of introducing various biases into the regression model when analyzing HAP cases. Column 1 shows the ‘correct’ model, whereas column 2 shows the effect of ignoring the time dependency of exposure. Controls are matched to cases within the same main diagnosis group (ICD-10) to control for cost clustering, but no time-to-exposure matching is applied. The estimated incremental cost effect increases to €30,500, with reimbursements estimated to be €29,500. Extra length of stay increases more than twofold compared to the correct model.

The third column shows the results of a regression controlling for overall length of stay instead of matching on the time of infection. Here, estimates are lower than in the correct model. While length of stay adjustment corrects the overestimation caused by the time-dependent bias when ignoring the time-varying nature of hospital-acquired infections, it underestimates the true effect. Since increased length of stay is a result of a hospital-acquired infection controlling for it ignores part of the true effect by conditioning on the future.

Table 5 shows the burden of *Pseudomonas aeruginosa* pneumonia stratified for multidrug-resistance. Interestingly, the most pronounced effect of multidrug-resistance was in community acquired cases. Although the number of MDR-HAPs is rather low (*n* = 15), there only seem to be slight differences (see Fig. 2) in the time points of exposure between non-MDR-HAP and MDR-HAP cases (14.4 days and 21.4 days, *p* = 0.088). See also Additional file 1: Figure S1 and S2 for details of the comparison between (MDR-) HAPs and uninfected controls. However, the substantial differences in total in-hospital costs underline the need for the within-main-diagnosis-time-to-exposure stratification approach applied that allows for appropriate treatment of time-dependent exposure, while also accounting for the clustering of costs within main diagnosis groups.

**Table 5:** Burden of *Pseudomonas aeruginosa* pneumonia stratified for multidrug-resistance

	Hospital-acquired cases (HAP)		Community-acquired cases (CAP) where the infection was not the main diagnosis		Community-acquired cases (CAP) where the infection was the main diagnosis	
	Non-MDR-HAP (*N* = 126)	MDR-HAP (*N* = 15)	Non-MDR-CAP (*N* = 48)	MDR-CAP (*N* = 15)	Non-MDR- CAP (*N* = 27)	MDR –CAP (*N* = 5)
Additional costs (in €)	19,448.3^***^	12,875.3	4051.7^***^	27,251.06^***^	6121.2^***^	12,981.7
	[12,472.7, 26,423.9]	[− 3890.0, 29,640.6]	[1971.6, 6131.7]	[7732.4, 46,769.6]	[2657.6, 9584.8]	[−25,078.8, 51,042.2]
Additional reimbursement (in €)	17,729.8^***^	4728.6	2647.5^***^	18,070.3^***^	5077.4^***^	7676.6
	[9943.0, 25,516.7]	[− 7346.2, 16,803.5]	[1196.5, 4098.6]	[12,582.8, 23,557.7]	[1979.8, 8175.0]	[−30,929.8, 46,283.1]
Additional length of stay (in days)	9.524^***^	6.693^**^	5.102^***^	15.483^***^	9^***^	25
	[6.494, 12.554]	[1.532, 11.854]	[2.498, 7.705]	[10.490, 20.477]	[5.302, 12.697]	[−10.087, 60.087]
N	555		262		32	

95% confidence intervals in brackets, results of median regression

^*^ *p* < 0.1, ^**^ *p* < 0.05, ^***^ *p* < 0.01

Results of multivariate quantile regressions including a case-control variable as well as CCI, sex, age, and age^2^ as fixed effects (HAP and CAP) and MDR-status as effect modifier. For HD-CAP cases univariate quantile regression was used

## Discussion

*Pseudomonas aeruginosa* pneumonia is associated with substantial additional hospitalization costs which, on average, are not fully recovered through higher reimbursements. The additional costs generated are highest for hospital-acquired cases. Our sensitivity analysis for hospital-acquired cases showed that ignoring or incorrectly adjusting for the time-dependency substantially biases results. The extent of this bias underlines the need to carefully address time-varying exposure and to collect and provide data on infection onset. Overall, the proportion of MDR *Pseudomonas aeruginosa* is low (15%); and a pronounced effect of multidrug-resistance was detected for community acquired cases.

Our study has several limitations. First, being a single center study, the generalizability of the estimated effects may be limited. However, since the cost calculation method is standardized and is used by most German hospitals, the effects at other German hospitals should be comparable. Another limitation concerns the definition of time-at-risk for acquiring pneumonia. A large proportion of hospital-acquired pneumonia cases, particularly with *Pseudomonas aeruginosa*, are associated with mechanical ventilation and/or intubation. Since mechanical ventilation or intubation alters the likelihood of contracting pneumonia, ventilation-associated pneumonia (VAP) cases are usually compared to controls that are at risk, i.e. also ventilated [40]. This left truncation means that cases and controls should “enter the study” after ventilation has commenced. Attributing length of stay before onset of ventilation to the burden of VAP leads to length bias, which can overestimate the true effect. Since our dataset and routine data in general do not provide information on the time point of ventilation we are unable to take this into account, meaning that our results potentially overestimate the true effect.

Another important limitation is that we cannot rule out that the very large cost differences between HAP and CAP we found might be due to outliers, since MDR cases are rare (*N* = 15).

An additional limitation lies in the calculation of extra costs of community onset cases that are the primary reason for hospitalization. Setting the additional costs of pneumonia equal to the total costs of hospitalization assumes that patients would not have been admitted to the hospital without the condition. However, the extra costs may include costs unrelated to *Pseudomonas aeruginosa-* related pneumonia but which are attributable to the treatment of comorbidities unrelated to the main diagnosis (e.g. a patient with a chronic condition is more expensive than one without). The full costs of hospitalization may therefore be an overestimate. It can however be argued that these are societal costs, and that from the hospital perspective the counterfactual is that those secondary diagnosis conditions would not have led to hospital admission.

## Conclusions

For future research, we wish to stress the importance of considering the time-dependent aspects of incremental costs to provide reliable estimates for accurate evaluation of the potential effectiveness of different interventions. In addition, incremental costs should be calculated giving adequate consideration of the risks at baseline and with carefully chosen controls. Our estimates provide a first detailed estimation of the additional costs of *Pseudomonas aeruginosa* pneumonia in German hospital settings and can guide further research by quantifying the extent of the different biases these analyses are prone to.

## Supplementary information

**Additional file 1: Figure S1.** HAP controls only. **Figure S2.** MDR-HAP: cases and controls.

## Data Availability

Use of the data outside the Freiburg University Medical Center IT network is restricted, so it unfortunately cannot be made available.

## Abbreviations

CAP: Community-acquired pneumonia
CCI: Charlson Comorbidity Index
HAI: Healthcare-associated infection
HAP: Hospital-acquired pneumonia
MDR: Multidrug-resistant, multidrug-resistance
VAP: Ventilation-associated pneumonia
