# Supplementary material for: Measuring the in-hospital costs of Pseudomonas aeruginosa pneumonia: methodology and results from a German teaching hospital
Source: BMC Infect Dis. 2019 Dec 3;19:1028. doi: 10.1186/s12879-019-4660-5 (PMC6888947; doi:10.1186/s12879-019-4660-5)

**Measuring the in-hospital costs of *Pseudomonas aeruginosa* pneumonia: Methodology and results from a German teaching hospital – Additional files**

*Contact: kaier@imbi.uni-freiburg.de*

**Figure S1: HAP controls only**

C

**
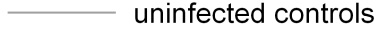
**

**Figure S2: MDR-HAP: cases and controls**

**
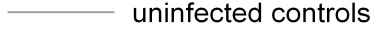
**


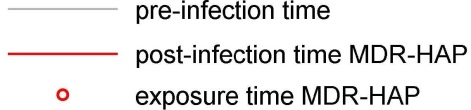

Supplement: Supplementary file 1 — Additional file 1: Figure S1. HAP controls only. Figure S2. MDR-HAP: cases and controls. [file 12879_2019_4660_MOESM1_ESM.docx]
